# Sorption of Strontium to Uraninite and Uranium(IV)–Silicate Nanoparticles

**DOI:** 10.1021/acs.langmuir.1c02927

**Published:** 2022-02-28

**Authors:** Thomas S. Neill, Katherine Morris, Carolyn I. Pearce, Nicholas K. Sherriff, Nick Bryan, Bruce Rigby, Samuel Shaw

**Affiliations:** † Research Centre for Radwaste Disposal and Williamson Research Centre, School of Earth & Environmental Sciences, The University of Manchester, Oxford Road, Manchester M13 9PL, U.K.; ‡ Pacific Northwest National Laboratory, Richland, Washington 99354, United States; § National Nuclear Laboratory, Chadwick House, Warrington Road, Birchwood Park, Warrington WA3 6AE, U.K.; ∥ Sellafield Ltd., Hinton House, Birchwood Park Avenue, Risley, Warrington, Cheshire WA3 6GR, U.K.

## Abstract

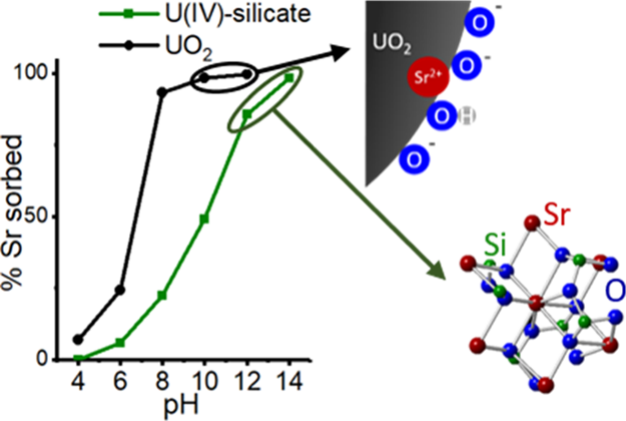

Spent nuclear fuel contains both uranium (U) and high yield fission products, including strontium-90 (^90^Sr), a key radioactive contaminant at nuclear facilities. Both U and ^90^Sr will be present where spent nuclear fuel has been processed, including in storage ponds and tanks. However, the interactions between Sr and U phases under ambient conditions are not well understood. Over a pH range of 4–14, we investigate Sr sorption behavior in contact with two nuclear fuel cycle relevant U(IV) phases: nano-uraninite (UO_2_) and U(IV)–silicate nanoparticles. Nano-UO_2_ is a product of the anaerobic corrosion of metallic uranium fuel, and UO_2_ is also the predominant form of U in ceramic fuels. U(IV)–silicates form stable colloids under the neutral to alkaline pH conditions highly relevant to nuclear fuel storage ponds and geodisposal scenarios. In sorption experiments, Sr had the highest affinity for UO_2_, although significant Sr sorption also occurred to U(IV)–silicate phases at pH ≥ 6. Extended X-ray absorption fine structure (EXAFS) spectroscopy, transmission electron microscopy, and desorption data for the UO_2_ system suggested that Sr interacted with UO_2_ via a near surface, highly coordinated complex at pH ≥ 10. EXAFS measurements for the U(IV)–silicate samples showed outer-sphere Sr sorption dominated at acidic and near-neutral pH with intrinsic Sr-silicates forming at pH ≥ 12. These complex interactions of Sr with important U(IV) phases highlight a largely unrecognized control on ^90^Sr mobility in environments of relevance to spent nuclear fuel management and storage.

## Introduction

Over 60 years of nuclear research and power generation has resulted in a significant global nuclear legacy for decommissioning and disposal. Uranium (U) is typically the most abundant element by mass in spent nuclear fuel (SNF).^1,2^ Additionally, high yield, high specific activity fission products (e.g., ^90^Sr and ^137^Cs) currently dominate the radioactivity inventory of SNF and other radioactive wastes.^3^^90^Sr (half-life 29 years) is a high-energy beta emitter and a significant contributor to dose in many nuclear fuel cycle scenarios. Understanding ^90^Sr speciation under relevant conditions is essential for the safe management and decommissioning of nuclear legacy facilities. Despite the colocation of ^90^Sr and U in many SNF storage and handling facilities, the interactions of Sr with U phases under ambient temperature and pressure conditions are poorly constrained. Understanding Sr sorption capacity, and mechanism(s) for Sr uptake onto key U phases present in SNF, is essential for predicting the behavior of ^90^Sr in these environments.

^90^Sr and U are colocated in nuclear fuel ponds including the legacy ponds and silos at the Sellafield nuclear facility, which are some of the most hazardous nuclear facilities in the UK.^4^ The nuclear fuel pond facilities are maintained at an elevated pH (11.5) to minimize fuel corrosion; however, historic issues have led to extensive corrosion of the Magnox nuclear fuel within the ponds.^5−7^ The water in these ponds is in contact with the cementitious material and therefore contains dissolved silicate. The corroded Magnox consists of Mg-rich corroded fuel cladding, corroded metallic U, fission products, and transuranics and is referred to as corroded Magnox sludge (CMS). CMS is a complex mix of brucite (Mg(OH)_2_) and other Mg–carbonate, hydroxide, and Mg–Al–Si hydroxides; SNF; and corroded metallic U.^8^ Iron sulfide minerals have also been identified in CMS, suggesting strongly reducing zones. Under these conditions, the corrosion of metallic U produces UO_2_,^8−11^ which has been observed during CMS characterization and metallic U corrosion experiments.^8,9^ Many other forms of nuclear fuel use UO_2_ as a fuel matrix,^2^ making UO_2_ abundant in most SNF storage scenarios. Importantly, under groundwater conditions (pH 8.4, 41 ppm Si), corrosion of metallic U in SNF can lead to the formation of U(IV) colloids.^11,12^ While the nanoparticulate structure of these colloids was initially identified as UO_2_, they show remarkably similar properties to silicate-stabilized UO_2_ colloids.^9^ These colloids also show similar properties to U(IV)–silicate colloids, which are stable under conditions related to SNF storage.^13,14^ Furthermore, recent work indicates that UO_2_ colloidal particles in many storage and disposal environments may have a silicate coating.^9^ The mobility of these relatively newly identified U(IV)–silicate colloidal phases is predicted to be high. In turn, this could impact the mobility of associated radionuclides, including ^90^Sr, during effluent treatment of waste streams originating from SNF storage facilities. Thus, the interaction of ^90^Sr with both UO_2_ and U(IV)–silicate colloids is of potentially high relevance to fuel pond storage and radioactive effluent treatment systems.

The behavior of Sr at circumneutral pH is dominated by reversible adsorption of the soluble Sr^2+^ ion to a range of solid phases as an outer-sphere complex.^15−19^ As the pH increases above the point of zero charge (pH_pzc_) of the solid phase, a net negative surface charge occurs, which results in positively charged Sr^2+^ having an increased affinity for the surface. At pH >12.5, Sr forms inner-sphere complexes with clays, iron oxides, and sediments, which have been linked to the formation of the Sr(OH)^+^ species in solution at pH > 12.^15,16^ Sr mobility is also affected by solubility-limiting phases at high pH. In the presence of carbonates, strontianite (Sr(CO)_3_) can form,^20^ and in the presence of silicates, strontium silicates have also been observed.^21−23^ In addition, Sr readily substitutes for calcium in calcite (CaCO_3_)^24,25^ and calcium silicate hydrate (CSH) cement phases.^26,27^ CSH was shown to uptake Sr via bonding to silanol (−Si–O–H) groups,^27^ highlighting the potential significance of Sr–silicate interactions in controlling ^90^Sr behavior. The effect of hyperalkaline pH on sediments and Sr mobility has also been investigated.^28−30^ A previous work, where sediments were reacted with cement leachate for 1 year, resulted in Sr becoming bound via an inner-sphere complex to an aluminosilicate gel alteration phase after reaction at room temperature. At higher temperatures (70 °C), Sr was incorporated into a newly formed zeolite phase.^28^

There have been several investigations into Sr uptake by colloids, including clay,^31^ silica,^32^ and natural groundwater^33^ colloids. Here, Sr was sorbed to the colloidal matter and, as with bulk solids at circumneutral pH, the sorption was reversible in most cases, suggesting that the outer-sphere sorption dominated. Furthermore, in most cases, the colloids did not significantly enhance the transport of Sr. In fact, retardation of Sr mobility occurred due to aggregation of colloidal particles, leading to immobilization of both the suspended phase and the sorbed Sr.^33^ There is a paucity of information on Sr–colloid interactions under the high pH conditions expected in both SNF ponds and intermediate level radioactive waste disposal. Previous Sr sorption studies at high pH suggest that more varied Sr–substrate interactions can occur.^15,16^

In terms of Sr interactions with U phases, data are sparse. Sr shows a strong affinity for sorption onto uranyl peroxide (studtite, UO_4_) and uranophane (a U(VI)–silicate, Ca(UO_2_)_2_(SiO_3_OH)_2_·5H_2_O).^34,35^ In alkaline SNF storage, Sr has a high affinity for substrates such as monosodium titanate (NaHTiO_5_).^36^ Sr also sorbs onto TiO_2_ particles in SNF storage ponds, with extended X-ray absorption fine structure (EXAFS) data suggesting some incorporation into the TiO_2_ structure.^37^ To the authors’ knowledge, no studies have been performed on Sr sorption to U(IV) phases. The interaction of UO_2_ with Sr has been well studied in relation to SNF due to the formation of ^90^Sr in UO_2_ fuel matrices during nuclear fission. SrO is known to form a solid solution with UO_2_ at high temperatures and pressures relevant to nuclear reactors.^38,39^ Additionally, uraninite ores (nominally UO_2_) are known to contain high amounts of Ca^2+^ and other divalent cations, including Sr^2+^ at concentrations above 200 ppm.^40,41^ However, there is a paucity of data on Sr interaction with UO_2_ or U(IV)–silicates under the low-temperature aqueous conditions relevant to SNF pond storage and intermediate level radioactive waste management.

In this study, sorption of Sr onto well-characterized nano-UO_2_ precipitates and nano-U(IV)–silicate colloids and precipitates was investigated across a range of pH (3.7–12 and 4–14, respectively). Ultrafiltration was used to assess the extent of absorption onto both colloidal and precipitated phases. Acid leaching experiments, in combination with transmission electron microscopy (TEM), provided further information on Sr–UO_2_ interactions. Select samples were analyzed by EXAFS spectroscopy to probe the mechanisms of Sr sorption onto these two key phases. These results offer new insights into Sr–U(IV) interactions, which have implications for ^90^Sr mobility in nuclear decommissioning and waste management scenarios.

## Methods

Triplicate experiments were carried out under a N_2_/H_2_ atmosphere (<20 ppm O_2_) with solutions prepared under anaerobic conditions using deoxygenated deionized water (18 MΩ). Nano-particulate UO_2_ was prepared by the dilution of a U(IV)–carbonate solution (20 mM) in deionized water at a 1:19 ratio.^13,14^ U(IV)–silicate samples were prepared by a 1:19 dilution of a 20 mM U(IV)–carbonate solution with 4.2 mM sodium metasilicate Na_2_SiO_3_.^13,14^ The pH of the UO_2_ and U(IV)–silicate samples was then lowered to pH 4 by titration with hydrochloric acid (1.5 M HCl) with stirring for 30 min to degas CO_2_. This was done to avoid Sr–carbonate formation during the experiments. The pH was then adjusted to target values [pH 4, 6, 8, 10, 12, and 14 for U(IV)–silicate experiments and 3.7, 6, 8, 10, and 12 for UO_2_ experiments] with 1.5 M NaOH. The ionic strength of the experiments (pH 3.7–12) was controlled to 0.15 M by the addition of NaCl. Experiments were pre-equilibrated for 7 days with pH recorded throughout, and then 0.05 M SrCl_2_ was spiked to yield a Sr concentration of 0.058 mM in solution. Samples were equilibrated for a further 7 days before analysis.

Sr sorption to UO_2_ and U(IV)–silicate phases was assessed using centrifugation–ultrafiltration at 8000*g* for 12 min using polyethersulfone (PES) filters (3 kDa, ∼1.5 nm^13,42,43^) and syringe filtration (0.22 μM, PES filters). U and Sr in the <1.5 nm size fraction were classified as “in solution”, 1.5–220 nm particles were classified as “colloidal”, and >220 nm particles were classified as “precipitates”. Total U and Sr in the filtrates were measured by inductively coupled plasma–mass spectroscopy (ICP–MS) (Agilent 7500cx), and in the U(IV) silicate systems, total silicon was measured using ICP-atomic emission spectroscopy (PerkinElmer Optima 5300 dual view).

To assess the nature of the binding of Sr to UO_2_, acid leaching experiments were performed on Sr sorbed to UO_2_ at pH 12, 10, 8, and 3.7. After 7 days of equilibration with Sr, the solution pH was decreased to 3.2 (±0.1) in all samples. Samples were equilibrated at pH 3.2 and filtered after 24 h using centrifugation–filtration (3 kDa, 1.5 nm PES filters), and the total Sr and U were analyzed in filtrates using ICP–MS. The pH was then further reduced to pH 2 and 3 kDa ultrafiltration, and ICP–MS measurements were repeated after a further 24 h.

TEM samples for select Sr/UO_2_ experiments (pH 8 and 12) were mounted on carbon-coated copper TEM grids (Agar Scientific) and imaged using an FEI TF30 analytical FEG transmission electron microscope.

For Sr K-edge EXAFS analysis, in colloid free systems, samples were centrifuged (7500*g*, 10 min) to isolate solids from the solution and mounted in anaerobic cells under a N_2_/H_2_ atmosphere. In U(IV)–silicate experiments at pH 6, 8, and 10 where colloids persisted, solid samples for XAS analysis were collected by centrifugation–filtration on 3 kDa PES filters.^14^ The resultant XAS samples had between 1000 and 10,000 ppm Sr present on solids. Samples were analyzed using the Diamond Light Source B18 beamline at the Sr K-edge using a Si(111) monochromator at liquid nitrogen temperature in the fluorescence mode. The data were analyzed using the Demeter software package containing Athena and Artemis, FEFF6. Finally, thermodynamic modeling of speciation was performed using PHREEQC with the ThermoChimie-TDB database (see the Supporting Information).^44^

## Results and Discussion

### U(IV) Phases

**Figure 1:**
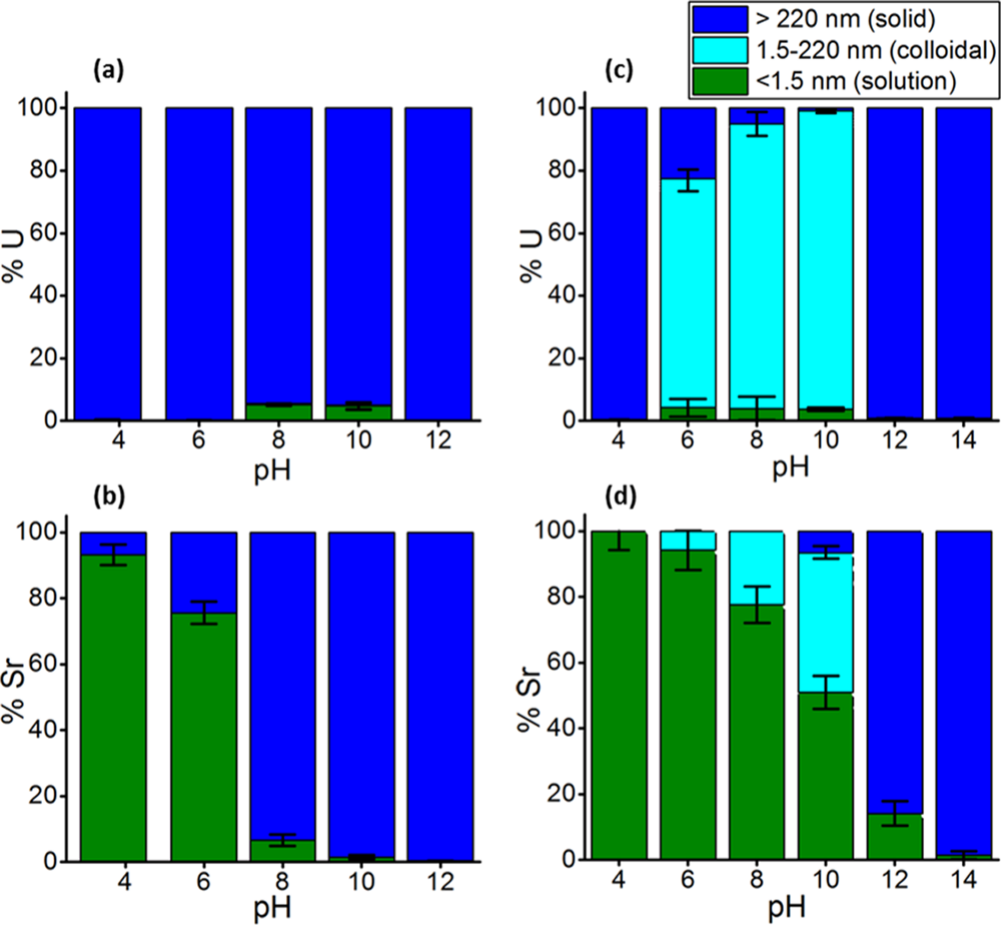
Filtration results for UO_2_ experiments showing size distribution of uranium species (a) and strontium (b) and U(IV)–silicate experiments showing size distribution of uranium species (c) and strontium (d). Note: U and Sr present in the <1.5 nm fraction are assumed to be in true solution, 1.5–220 nm are colloidal, and >220 nm are precipitated (solid). 100% corresponds to the total initial concentration of the elements. Error bars represent 1 standard deviation on triplicate measurements.

In the UO_2_ systems, there was no evidence for colloidal uranium, with UO_2_ precipitates forming at pH 3.7–12 (Figure 1a,b). In U(IV)–silicate experiments, the U (Figure 1c) and Si (Figure S1) filtration data showed a strong correlation, suggesting that an intrinsic uranium silicate phase was formed under all conditions (pH 4–14). The molar Si/U ratio of these U(IV)–silicate phases in the colloidal and/or precipitate fraction (>1.5 nm) was 2.1 at pH 4 and increased to 2.4 at pH 6. The ratio then stabilized at 2.4 from pH 6 to 10 and dropped to 1.8 at pH 12 and 0.8 at pH 14 (Figure S2). Colloidal U(IV)–silicate particles, defined here as between 1.5 and 220 nm, were formed between pH 6 and 10, coincident with the highest Si/U ratios. In addition, a small fraction of dissolved U (<3 kDa fraction) was present between pH 6 and 10 for both the U(IV) silicate and UO_2_ systems. Overall, these observations are consistent with previous experiments.^13,14^ These results confirm that pH adjustment to pH 4 to facilitate CO_2_ degassing in the current experiments, necessary to prevent Sr–carbonate formation at high pH, had no significant effect on the colloidal behavior and composition of these phases.

### Sr/UO_2_ Filtration and Desorption Investigations

Sr sorbed onto UO_2_ across the experimental pH range (3.7–12), with sorption increasing with increasing pH (Figure 1a,b). At pH 3.7, only 7% of Sr was removed from solution in the >220 nm fraction. This increased to 24% removal of Sr in the >220 nm fraction at pH 6, 93% at pH 8, 99% at pH 10, and >99% at pH 12. There was no evidence for colloidal (1.5–220 nm) Sr in any of the UO_2_ experiments, consistent with Sr sorbed onto the UO_2_ precipitate.

More Sr was removed from solution in UO_2_ systems, compared to that from U(IV)–silicate systems at the same pH, indicating that Sr has a higher overall affinity for UO_2_ (Figures 1 and S3). The increasing Sr removal from solution with increasing pH in the presence of UO_2_ can be explained in part by the changing UO_2_ surface charge. Sr solution speciation is dominated by the hydrated Sr^2+^ ion at pH < 12; thus, below the pH_pzc_ of UO_2_ (5.8), the surface is positively charged and Sr sorption would be low due to electrostatic repulsion.^45^ With increasing pH, the UO_2_ surface becomes more negative, increasing the affinity for the positively charged Sr^2+^ ion. While the trend for increasing Sr sorption at higher pH was anticipated, the magnitude of Sr sorption onto UO_2_ observed here is notably higher at pH 8 and 10 than that in previous studies on Sr sorption to U(VI)–peroxide;^35^ CSH;^26^ and Fe(III) oxyhydroxides, clays, and sediments.^15^ When the calculated distribution coefficients (*K*_d_) for Sr on UO_2_ were compared with those for U(IV)–silicates, and with other published values, UO_2_ showed a significantly higher *K*_d_ for Sr than the other phases (Figure S4). Although this may be due in part to the high surface area of the nanoparticulate phases, the surface area differences for the UO_2_ and U(IV)–silicate used in this study are minimal given their similar primary particle sizes.^13,14^ Thus, it seems likely that the intrinsic affinity of Sr^2+^ for the UO_2_ surface is higher.

In order to further explore the nature of the Sr binding to UO_2_, desorption experiments, with acid leaching, were carried out on the pH 3.7, 8, 10, and 12 samples. A pH of 3.2 was selected to target surface-bound Sr, as UO_2_ solubility is very low at this pH, and any Sr incorporated in the bulk, crystalline UO_2_ would remain associated with the solid phase. Data from the acid leaching pH 3.2 24 h desorption step showed that essentially all of the Sr was rereleased into the solution for all starting pH conditions (Figure S5). Under these leaching conditions, <0.5% of U was in solution in all systems, confirming that there was minimal dissolution of UO_2_. This confirms that Sr was labile in all the UO_2_ sorption experiments and was likely associated with the surface or near-surface region of the UO_2_ particles. This suggests that Sr was not incorporated into the bulk structure of the nano-crystalline UO_2_ particles, where it would be expected to be more resistant to the pH 3.2 acid leaching conditions.

### Sr/UO_2_ Interaction Mechanisms

**Figure 2:**
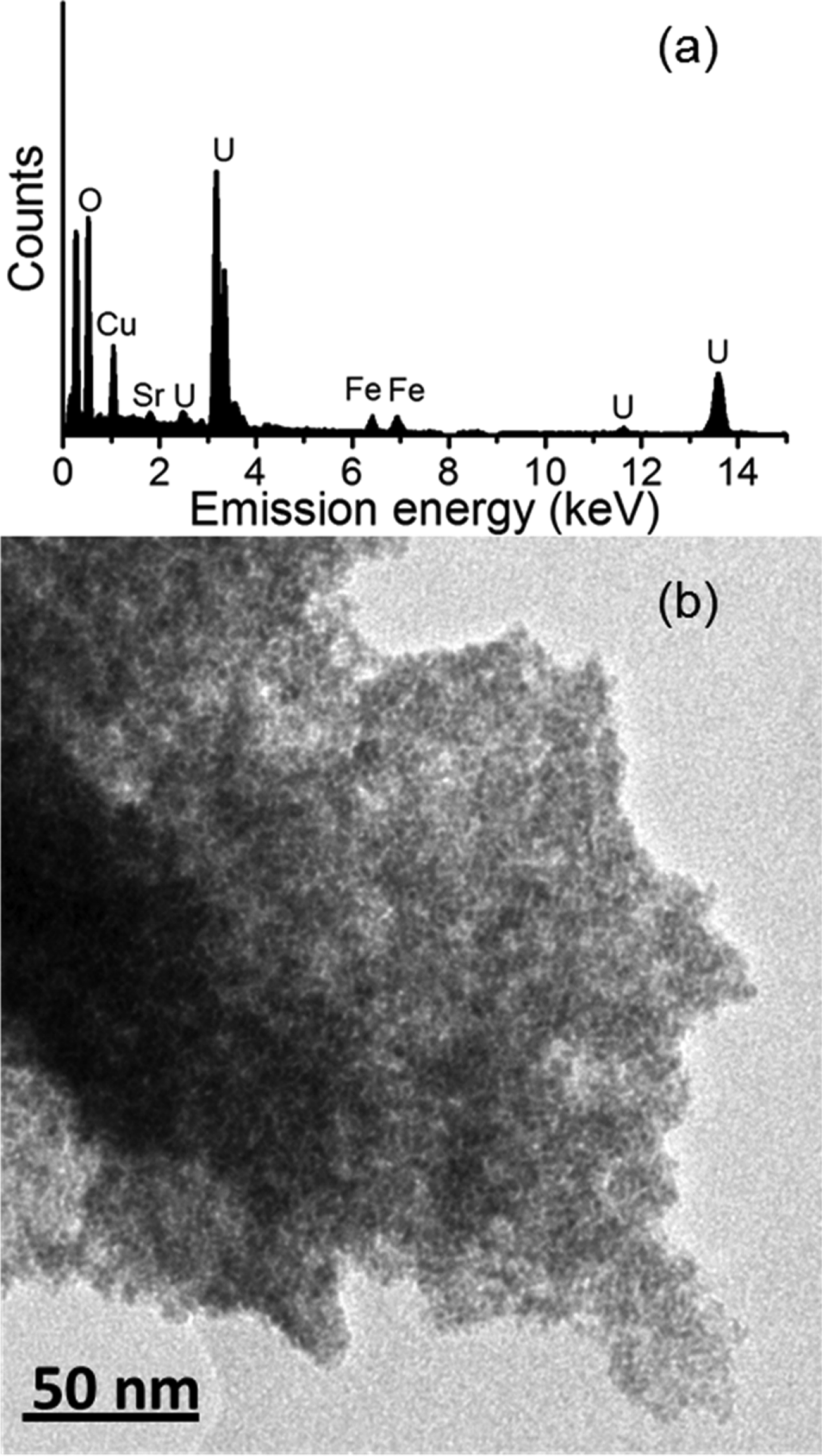
TEM image of UO_2_ particles formed at pH 12 with Sr associated (a) and EDX spectrum showing colocation of Sr and U (b) (Fe peaks are background features).

TEM imaging (Figure 2) confirmed that the nano-UO_2_ morphology was consistent with previous studies,^14,46^ and selected area electron diffraction (SAED) analyses confirmed the presence of UO_2_ (Figure S6). Here, energy-dispersive X-ray (EDX) spectroscopy showed that Sr was colocated with UO_2_ aggregates at pH 8 and 12. In both samples, there was no evidence for discrete Sr-rich phases from SAED or EDX analyses.

**Figure 3:**
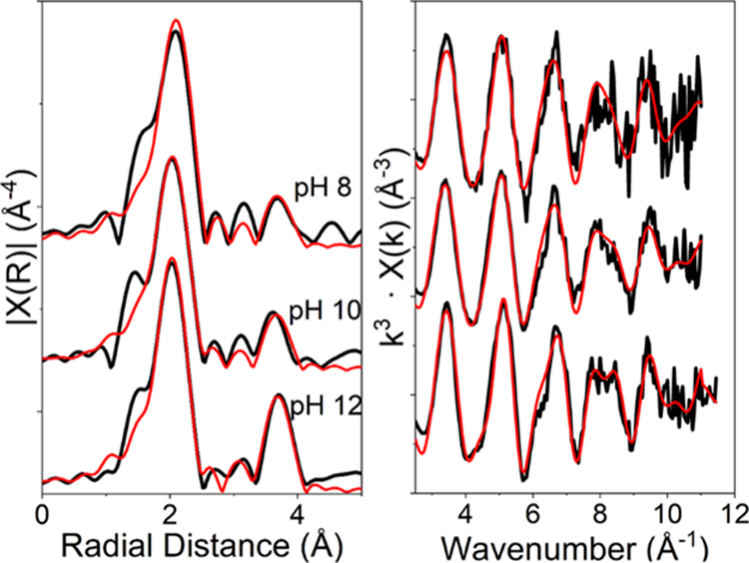
Sr K-edge EXAFS spectra (right) and Fourier transforms (left) for Sr bound to UO_2_ at pH 8, 10, and 12. The features at approximately 3.65 Å in the Fourier transform clearly suggest that Sr is not outer sphere bound and is instead in a more structured coordination environment.

Sr K-edge EXAFS data were also analyzed for the UO_2_ systems where almost complete Sr removal from solution had occurred at pH 8, 10, and 12. The EXAFS spectra and Fourier transforms are shown in Figure 3, with best fits for the data presented in Table 1. There was no evidence for SrCO_3_ formation in the EXAFS, confirming that CO_2_ ingress was minimal.^47^ For all three samples, there was evidence for significant long-range order in the samples with clear features in the Fourier transform at 3.5–4 Å. This differs from many previous EXAFS analyses on Sr sorption systems with other substrates where outer-sphere sorption dominates at pH ≤ 12 and no peaks in the Fourier-transformed Sr K-edge EXAFS spectra are observed at distances >2.5 Å.^15−18^ At pH 8, the EXAFS data were best fit with 9 O backscatterers at 2.63 Å. In addition, the fit was improved by the inclusion of a second shell of 1.5 U backscatterers at 3.67 Å. The presence of this second coordination shell fitted with U backscatterers suggests that Sr is in close proximity to the UO_2_ surface, consistent with it being sorbed as an inner-sphere complex. At pH 10, the EXAFS data could also be fitted with 9 O backscatterers in the first shell. However, the fit was improved by splitting the O shell, resulting in 2 Sr–O distances of 2.58 and 2.72 Å, with coordination numbers of 7.2 and 3.8, respectively, and with 1.8 U backscatterers at 3.65 Å. The fitting for the pH 12 system was also improved by similar splitting of the first O shell and 3.5 U backscatterers at 3.66 Å. Interestingly, there was a clear trend to increased coordination numbers for the U backscatterers with increasing pH from 1.5 to 3.5 backscatterers as pH increased from 8 to 12 (Table 1). This was indicative of a more structured local coordination environment developing for Sr as the pH increased. While the pH 8 EXAFS fit suggested formation of an inner-sphere sorption complex, the split O shell in the pH 10 and 12 EXAFS fits, along with the increased U coordination at 3.66 Å, indicated an alternative mechanism for the Sr–UO_2_ interaction. The splitting of the O shell observed in the best fits at pH 10 and 12 suggested that Sr was not incorporated into UO_2_ via substitution into the U^4+^ sites. Here, for the incorporated Sr^2+^, 8 coordinate Sr would be expected with Sr–O distances of almost 2.38 Å, which is in contrast with the best modeled fits (Table 1). Additionally, the Sr–U bond distance was fitted at approximately 3.66 Å for the pH 8, 10, and 12 samples, significantly shorter than the U–U distance in uraninite (3.87 Å).^48^ As Sr^2+^ has a larger atomic radius than U^4+^, this short Sr–U distance would not be expected for Sr-substituted UO_2_. These results are consistent with an alternative mechanism that may be referred to as “surface-incorporated” Sr^2+^ at pH 10–12, which is in good agreement with desorption results showing reversible Sr sorption. Overall, the chemical and spectroscopic data suggest that Sr is most likely to be surface incorporated, chemically (but also reversibly) bound in the disordered near-surface of the nano-UO_2_ and highlights an extensive interaction of Sr with UO_2_ in these systems.

**Table 1:** EXAFS Fit Data for Sr–UO_2_ Systems^a^

sample	path	*N*	*R* (Å)	σ^2^	Δ*E*_0_	*R*
pH 8	Sr–O	9	2.63(1)	0.009(1)	4.2(11)	0.023
	Sr–U	1.5	3.67(3)	0.009(4)		
pH 10	Sr–O.1	7.2	2.58(1)	0.008(2)	–2.6(10)	0.0078
	Sr–O.2	3.8	2.72(1)	0.009(5)		
	Sr–U	1.75	3.65(3)	0.010(2)		
pH 12	Sr–O.1	7	2.58(1)	0.006(1)	4.2(8)	0.0092
	Sr–O.2	4	2.74(3)	0.008(4)		
	Sr–U	3.8	3.66(2)	0.011(2)		

a Coordination numbers (*N*), U bond distances [*R* (Å)], Debye–Waller factors (σ^2^), shift in energy from the calculated Fermi level (Δ*E*_0_), and “goodness of fit” factor (*R*). Coordination numbers were fixed, and amplitude factors were fixed as 1. Numbers in parentheses are the standard deviation on the last decimal place.

This proposed surface incorporation mechanism for Sr is novel; however, Sr–U-oxides have been formed previously at high temperatures.^38,39,49^ Mixed Sr–U-oxides have been observed at *T* > 1000 K as either Sr^2+^ substituted into U sites within UO_2_ or via formation of a perovskite SrU(IV)O_3_ structure.^49,50^ Several studies have also shown negligible differences in the thermodynamics of formation of SrUO_3_ compared to those in the binary oxides UO_2_ and SrO, suggesting that SrUO_3_ formation is possible and perhaps thermodynamically favored in the presence of excess UO_2_.^51−54^ Additionally, in the current study, the high surface area of the nanoparticulate UO_2_ may promote the Sr reaction at the UO_2_ surface. The split shell and high O coordination number in the EXAFS fits for both pH 10 and 12 samples certainly suggest that the Sr coordination environment is very different from that of the absorbed Sr and is perovskite-like.^55,56^ Indeed, within the perovskite structure of Ca_1–*x*_Sr_*x*_TiO_3_, Sr has 12 O atoms at an average distance of 2.72 Å in the first coordination shell.^56^ Furthermore, the Sr–U distance of 3.66 ± 0.01 Å is also better represented with a perovskite-like structure and significantly different from the 3.87 Å U backscatterer expected in UO_2_.^48^ For example, the Sr–Ti distance in SrTiO_3_ is 3.36 Å^56^ and Sr–Zr distances in SrZrO_3_ at room temperature average 3.5 Å.^55^ Given the relative ionic radii follow the trend U^4+^ > Zr^4+^ > Ti^4+^, the Sr–U distance of 3.66 Å observed here seems credible for a SrUO_3_-like environment and is significantly different from the U–U distance found in uraninite at 3.87 Å, which would be expected for Sr incorporated into UO_2_. EXAFS fitting for the pH 10 and pH 12 samples showed Sr–U coordination numbers of 1.5–3.8, much lower than those expected if Sr was held in a crystalline Sr–U-oxide coordination environment (e.g., Sr–Ti coordination in a comparable structure, SrTiO_3_, is 8).^56^ Additionally, a Sr backscatterer indicating significant Sr–Sr coordination which would be expected in a Sr–U-oxide phase was not observed in the EXAFS fitting. Finally, TEM imaging and SAED analysis did not indicate any intrinsic Sr–U-oxide phase, with Sr apparently associated with nano-UO_2_.

Overall, the most likely speciation for Sr is surface incorporation of Sr on UO_2_, resulting in a perovskite-like Sr local coordination environment at the disordered UO_2_ surface. This is consistent with the EXAFS fitting, which showed evidence for Sr–U coordination across the fits at pH 8, 10, and 12, TEM showing that Sr was colocated with UO_2_, and acid leaching experiments indicating that Sr was labile in pH 8, 10, and 12 experiments. Given the clear trend of an increase in U backscatterers in the Sr EXAFS fits with increasing pH, the Sr reaction with the surface appears to be pH-dependent. Sr is in a less structured inner-sphere complex at pH 8 and in a more structured environment at pH 12. A near-surface incorporation could also explain the elevated Sr–U coordination in the EXAFS fit for the pH 12 system (3.8), despite Sr remaining labile in the acid leaching experiment. The Sr–UO_2_ reaction appears to be highly pH-dependent according to the acid leaching experiment, where a significant decrease in pH led to almost complete removal of Sr. Given only approximately 1% Sr by weight was sorbed to the UO_2_ particles, we suggest that Sr would be unlikely to significantly alter the bulk structure of UO_2_ as Sr complexation would occur primarily in the poorly ordered near-surface region of the UO_2_ nanoparticles.

### Sr/U(IV)–Silicate Interactions

In the U(IV)–silicate sorption experiments, the removal of Sr from solution increased with increasing pH (Figure 1c,d). No Sr removal was observed at pH 4. At pH 6, 6% of Sr was removed from solution and was present in the colloidal size fraction and therefore likely associated with the 73% of U which was present as the U(IV)–silicate colloid. The amount of Sr in the colloidal size fraction steadily increased to 22% at pH 8. At pH 10, 43% of Sr was in the colloidal size fraction, with 5% in the aggregated >220 nm fraction. At pH 12, 86% of Sr was associated in the aggregated >220 nm fraction and, at pH 14, 99% of Sr was in the >220 nm aggregated fraction. At pH 4, 12, and 14, no colloidal U(IV) was present. Colloidal Sr was not observed when there was no colloidal U, confirming that Sr was associated with the U(IV)–silicate colloids and did not form intrinsic Sr colloids.

At pH ≤ 10, increasing Sr sorption with increasing pH was attributed to an increasing negative surface charge of the U(IV)–silicate between pH 4 and 10; U(IV)–silicate particles, with a pH_pzc_ of approximately 4–4.5, will have a positive charge at low pH which becomes negative at pH > pH_pzc_.^13^ The presence of a significant fraction of colloid-associated Sr when U(IV) was also colloidal between pH 6 and 10 (Figure 1) suggests that U(IV)–silicate colloids may act as significant vectors for Sr. Indeed, a maximum of 43% of the total Sr was associated with colloidal material at pH 10, suggesting a significant control on Sr mobility. Although the extent of Sr removal from solution increases at pH > 10, Sr was associated with the precipitate and therefore immobilized. These results highlight that U(IV)–silicate may act as a colloidal vector for Sr between pH 6 and 10; however, at pH > 10, Sr mobility was reduced under these conditions.

### Sr/U(IV)–Silicate Interaction Mechanisms

**Figure 4:**
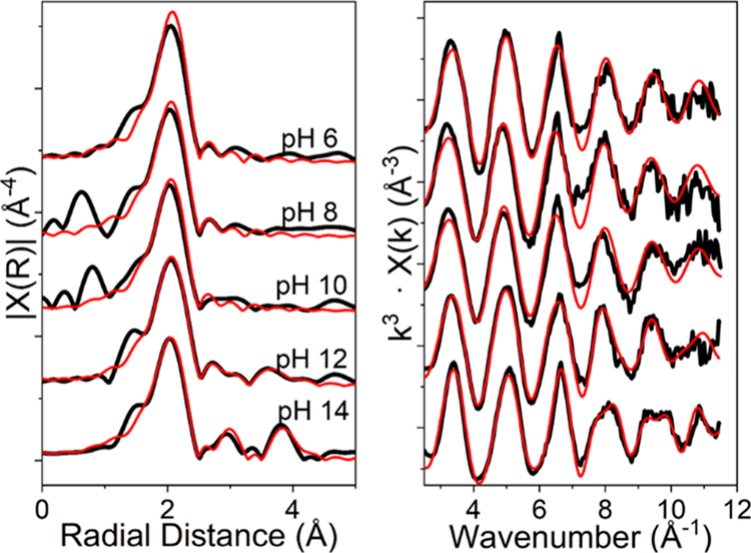
Sr K-edge EXAFS spectra (right) with accompanying Fourier transforms (left) for Sr bound to U(IV)–silicate at pH 6–14. Features at *R* + Δ*R* > 2.3 Å in the Fourier transforms indicate inner-sphere sorption and incorporation at pH > 10.

Sr EXAFS fits of the U(IV)–silicate systems (filtered colloids at pH 6, 8, and 10, and centrifuged solids at pH 12 and 14) are shown with corresponding best fits in Figure 4 and Table 2. Under all conditions, the first shell was fitted with 9 oxygen atoms at 2.61 Å. At pH 6, 8, and 10, there were no significant features in the Fourier transform beyond the first shell, and inclusion of any additional backscatterers in the models did not improve the fits. This confirmed that Sr sorption was predominantly outer sphere, similar to previous work on a range of environmental matrices at circumneutral pH.^15−18^ The outer-sphere sorption is also in agreement with colloidal transport studies of Sr, which showed Sr to be reversibly bound to colloidal particles at circumneutral pH.^31−33^ Overall, this suggests that when U(IV) is predominantly in colloidal form (pH 6–10), Sr will be sorbed as an outer-sphere complex, meaning that Sr is likely to be labile.

**Table 2:** EXAFS Fit Data for Sr–U(IV)–Silicate Systems^a^

sample	path	*N*	*R* (Å)	σ^2^	Δ*E*_0_	*R*
pH 6	Sr–O	9	2.61(1)	0.008(1)	0.7(10)	0.024
pH 8	Sr–O	9	2.61(1)	0.008(1)	–2.3(13)	0.011
pH 10	Sr–O	9	2.61(1)	0.009(1)	–2.3(11)	0.013
pH 12	Sr–O	9	2.61(0)	0.009(2)	–2.2(3)	0.0018
	Sr–Si	0.8	3.26(2)	0.009(2)		
	Sr–Sr	2	4.16(1)	0.012(2)		
pH 14	Sr–O	9	2.60(0)	0.010(1)	–2.8(11)	0.013
	Sr–Si.1	1.8	3.26(4)	0.014(6)^a^		
	Sr–Si.2	1.7	3.96(8)	0.014(6)^a^		
	Sr–Sr	4.5	4.28(3)	0.012(3)		
SrSiO_3_	Sr–O*	8	2.65			
	Sr–Si.1	4	3.34			
	Sr–Si.2	2	3.87			
	Sr–Sr*	6	4.12			

a Coordination numbers (*N*), U bond distances [*R* (Å)], Debye–Waller factors (σ^2^), shift in energy from the calculated Fermi level (Δ*E*_0_), and “goodness of fit” factor (*R*). Coordination numbers were fixed, and amplitude factors were fixed as 1. Numbers in parentheses are the standard deviation on the last decimal place. ^a^ indicates tied Debeye–Waller factors, and * indicates weighted average of similar paths. SrSiO_3_ structure from ref (57).

At pH 12 and 14, significant backscattering features were present at >2.3 Å in the Fourier transforms. This suggested that Sr was present in a more structured coordination environment compared to the pH 6–10 samples. Two likely scenarios were considered in the EXAFS fitting: (i) strong inner-sphere sorption to the U(IV)–silicate particles and (ii) formation of an intrinsic Sr silicate phase, for example, SrSiO_3_. Here, the pH 12 system could be fitted with Si and U shells (Table S2), suggesting the possibility of inner-sphere sorption/incorporation of Sr into U(IV)–silicate particles. However, a statistically improved fit (with lower *R*-factor) was achieved by fitting to a Sr silicate structure, implying the potential for a localized SrSiO_3_ structure (Table 2). This Sr silicate structure was fitted with low coordination numbers of 0.8 bidentate-bound (edge-sharing) Si at 3.26 Å and 2 Sr backscatterers at 4.16 Å, distances characteristic of SrSiO_3_.^57^ At pH 12, the Si and Sr coordination numbers in the fits were much lower than the those in the average bonding environment for SrSiO_3_ (Table 2), suggesting the presence of either a highly disordered structure and/or a mix of Sr–silicate and Sr sorption complexes. At pH 14, a Sr silicate-like structure also provided the best EXAFS fit, with a total of 3.5 Si backscatterers, 1.8 bidentate Si at 3.26 Å and 1.7 monodentate (corner-sharing) Si at 3.96 Å, and 4.5 Sr backscatterers at 4.28 Å. These two Sr–Si distances are very similar to the distances for edge- and corner-sharing SiO_4_ polyhedra, respectively, in SrSiO_3_ (3.34 and 3.87 Å).^57^ Although the Sr–Sr distance in the pH 14 fit (4.28 Å) was longer than the average Sr–Sr distance of 4.12 Å in SrSiO_3_ (Table 2), it is similar to the most distant Sr backscatterer at 4.30 Å (Table S1).

These data suggest that a disordered SrSiO_3_-like phase is contributing to the Sr coordination environment at pH 12 and 14. This is supported by previous investigations which confirmed Sr–silicate formation at high pH^21^ and reported greatly reduced Sr mobility and solubility in the presence of silicate.^22^ Thermodynamic modeling of the U(IV)–silicate/Sr system was also performed (Figure S7). Here, at pH > 11, modeling suggests that the solution would be oversaturated with respect to SrSiO_3_. These observations support the interpretation of the EXAFS fits, which show a disordered Sr silicate-like speciation at both pH 12 and 14.

## Conclusions

Two U(IV) phases, UO_2_ and U(IV)–silicates, demonstrate elevated Sr sorption under conditions relevant to SNF storage, contaminated land scenarios, and radioactive waste disposal where radioactive ^90^Sr and U will coexist. The high level of Sr sorption to UO_2_, as a surface-incorporated complex and the formation of Sr–silicate in the U(IV)–silicate systems suggest that these phases may play a critical role in controlling Sr mobility in contaminated environments, which has not been previously recognized. Insights into the mechanisms of Sr removal from solution suggest that there are complex interactions between Sr and both UO_2_ and U(IV)–silicate.

As some legacy spent fuel storage ponds at Sellafield are maintained at approximately pH 11,^58^ this study suggests that a large fraction of radioactive ^90^Sr^2+^ may be bound to the U(IV) phases present. At pH 10–11.5, U(IV)–silicates can be colloidal,^14^ suggesting that any associated Sr may be subject to enhanced mobility via sorption and colloidal transport. Solid Sr silicates are formed at pH > 12. Despite high Sr affinity for U(IV)–silicate colloids at pH ≤ 10, increased removal of Sr from solution occurred at pH 12 and 14 when no colloids were present. Therefore, U(IV)–silicates may facilitate or inhibit Sr transport, depending on the solution pH, and mobilization may be limited under highly alkaline conditions when these particles are not colloidal.

For UO_2_, Sr associations were dominated by a novel surface incorporation mechanism. While UO_2_ did not form colloids in this study, previous work has shown that UO_2_ can form colloids in the presence of silicates.^9,11^ Given that U(IV)–silicate colloids can form at alkaline pH, it is important to understand the impact of both UO_2_ and U(IV)–silicates on Sr behavior in order to predict Sr mobility where Sr and U are both present. Additionally, the surface incorporation in UO_2_ could result in less labile Sr at neutral/alkaline pH as desorption was only observed at acidic pH (<3.2).

## Supporting Information Available

Additional details on ultrafiltration results, dissociation constants, desorption investigations, STEM and XAS results, and thermodynamic modeling (PDF)
