# Supplementary material for: Sorption of Strontium to Uraninite and Uranium(IV)–Silicate Nanoparticles
Source: Langmuir. 2022 Feb 28;38(10):3090–7. doi: 10.1021/acs.langmuir.1c02927 (PMC9098169; doi:10.1021/acs.langmuir.1c02927)
Supplement: Supplementary file 1 — la1c02927_si_001.pdf [file la1c02927_si_001.pdf]

Supporting Information

Sorption of strontium to uraninite and uranium(IV)-silicate nanoparticles

Thomas S. Neill[a], Katherine Morris[a], Carolyn I. Pearce[b], Nicholas K Sherriff[c], Nick Bryan[c], Bruce Rigby[d], and Samuel Shaw[a]*

[a]Research Centre for Radwaste Disposal and Williamson Research Centre, School of Earth & Environmental Scineces, The University of Manchester, Oxford Road, Manchester M13 9PL, UK

[b]Pacific Northwest National Laboratory, Richland, WA 99354, USA

[c] National Nuclear Laboratory, Chadwick House, Warrington Road, Birchwood Park, Warrington WA3 6AE, UK

[d] Sellafield Ltd., Hinton House, Birchwood Park Avenue, Risley, Warrington, Cheshire, WA3 6GR, UK

*sam.shaw@manchester.ac.uk

This document contains 8 pages, 7 figures and 2 tables.

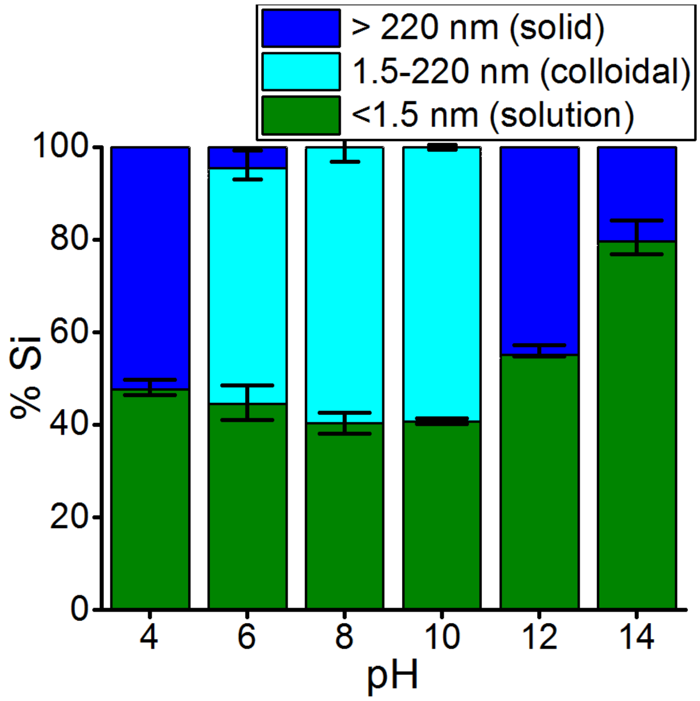

Figure S1: Filtration results for U(IV)-silicate experiments showing the size distribution of Si. Species below 1.5 nm in size are assumed to be in solution, 1.5-220 nm colloidal and > 220 nm are sedimented.

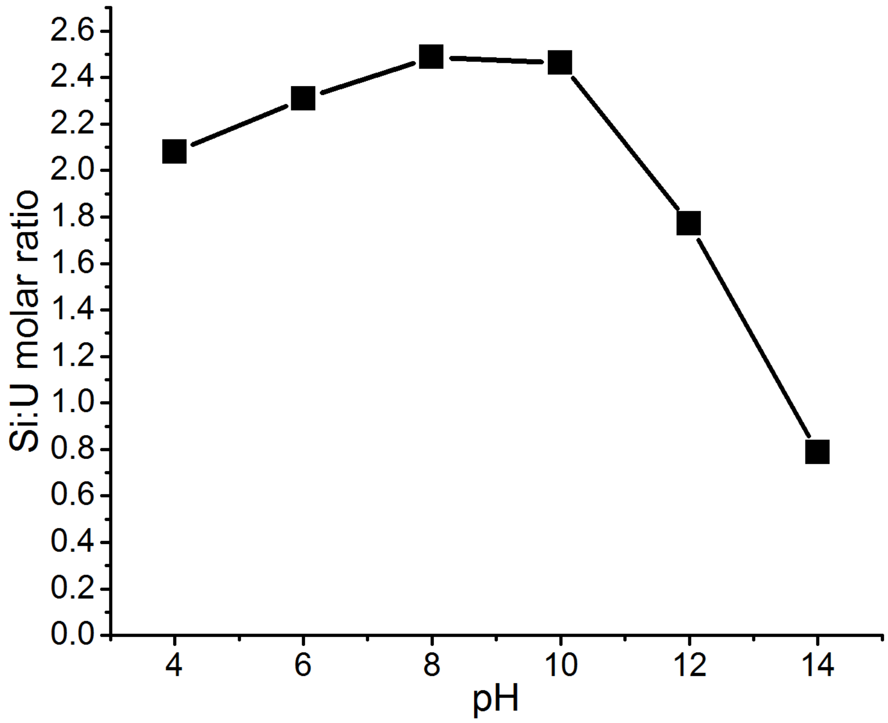

Figure S2: Si:U molar ratio taken from particulates >1.5 nm in U(IV)-silicate experiments. Figure shows increasing Si:U ratio at low pH, followed by subsequent decline in Si:U ratio at pH >8.

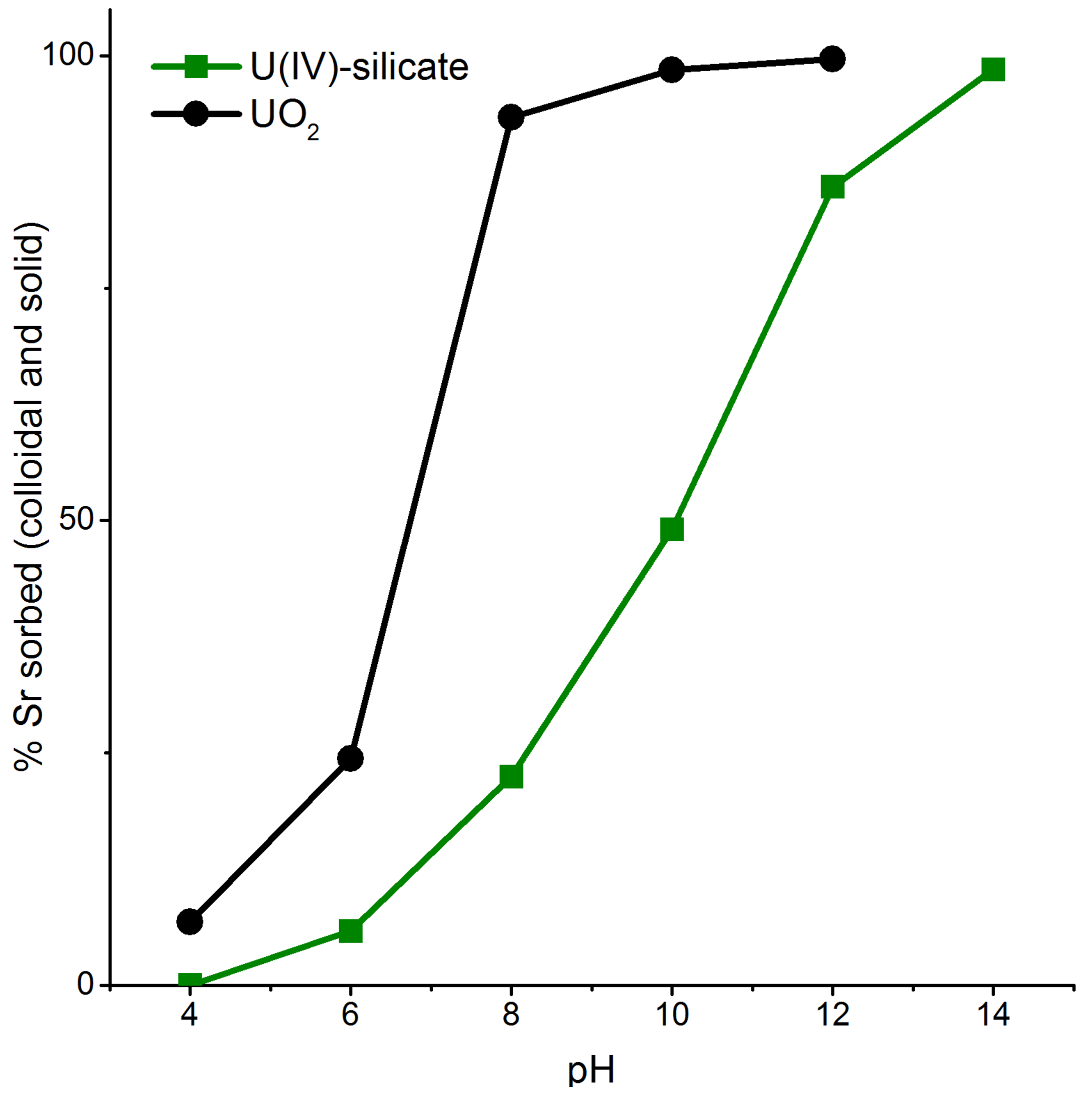

Figure S3: Comparison of the sorption of Sr in UO_2 and U(IV)-silicate systems showing higher sorption of Sr, at a lower pH, on UO_2.

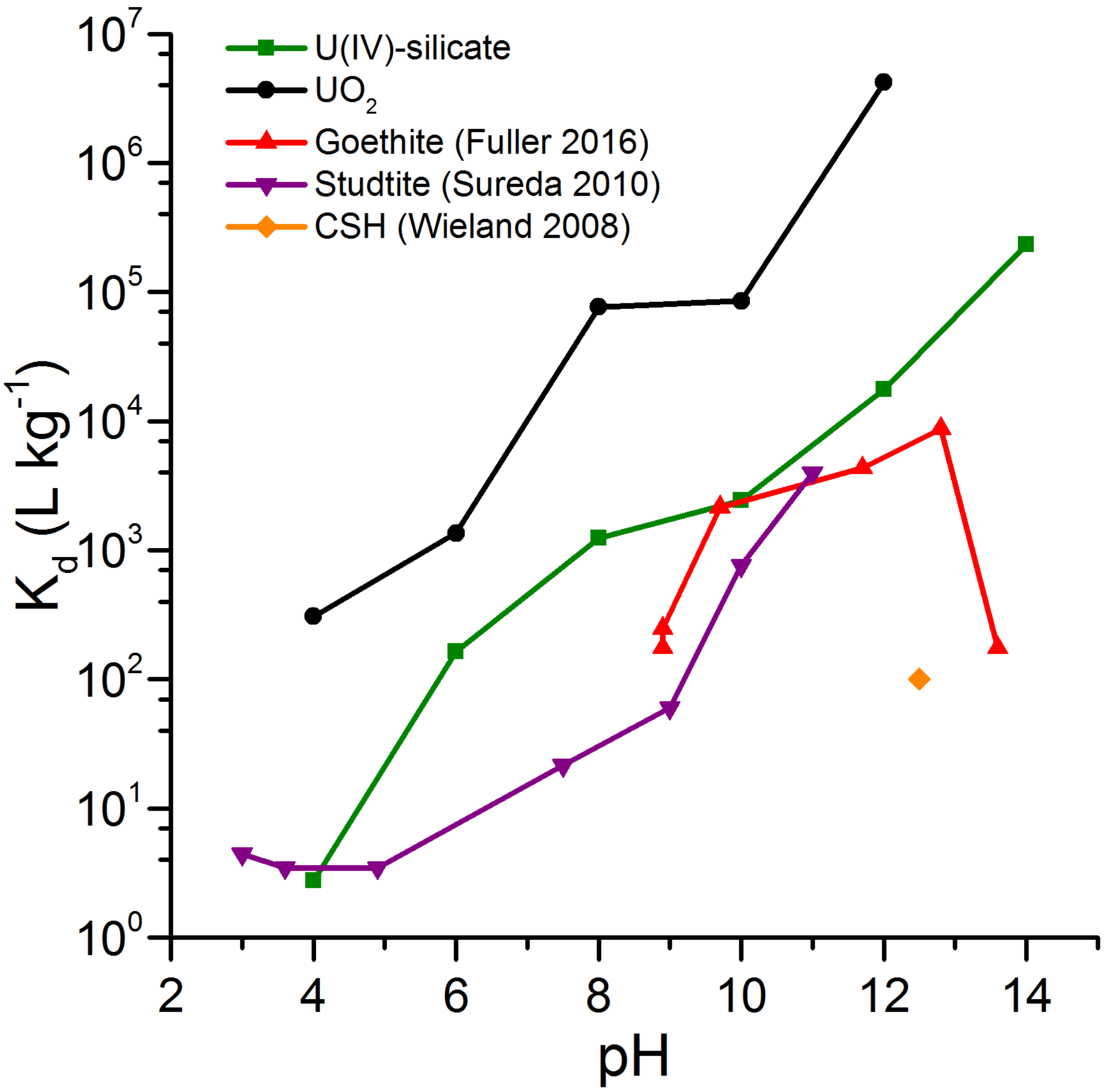

Figure S4: A comparison of dissociation constants (Kd) for Sr on U(IV)-silicate, UO_2 (both from this study), Goethite (Fuller *et al. (*2016)), Studtite (Sureda *et al.* (2010)) and CSH (Wieland *et al*. (2008)).

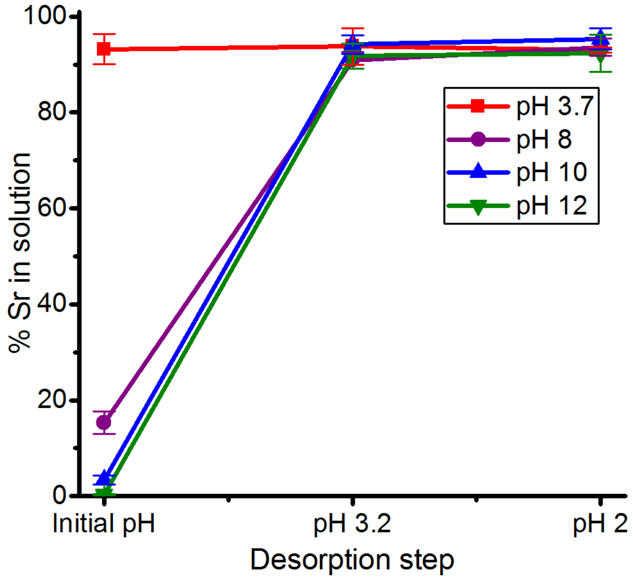

Figure S5: Results from Sr-UO_2 desorption experiments carried out at starting pH 3.7, 8, 10 and 12. In desorption experiments, the pH was reduced to 3.2 for 24 hours and the true solution (< 1.5 nm) sampled. The pH was then reduced to 2 for a further 24 hours and the true solution re-sampled to investigate the lability of Sr sorbed to UO_2.

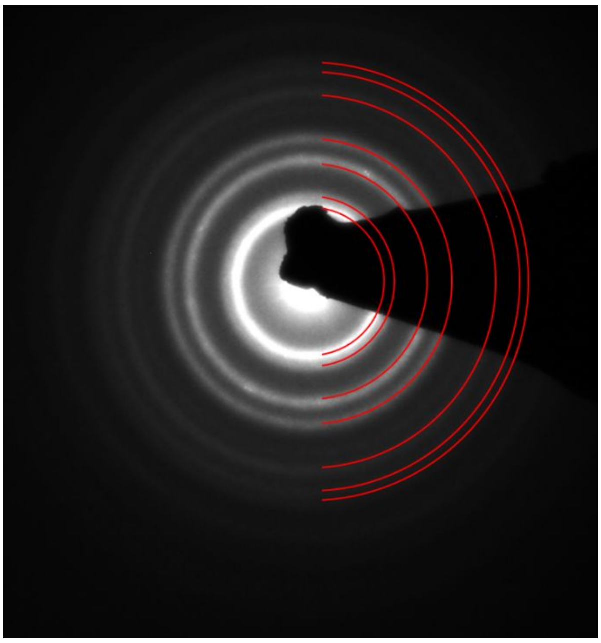

Figure S6: Selected area electron diffraction (SAED) of UO_2 particles formed at pH 12, with Sr incorporated. Red rings represent diffraction rings expected for UO_2.

Thermodynamic modelling

Thermodynamic modelling was performed using the PHREEQC software package and the specific ion theory (SIT) thermodynamic database.

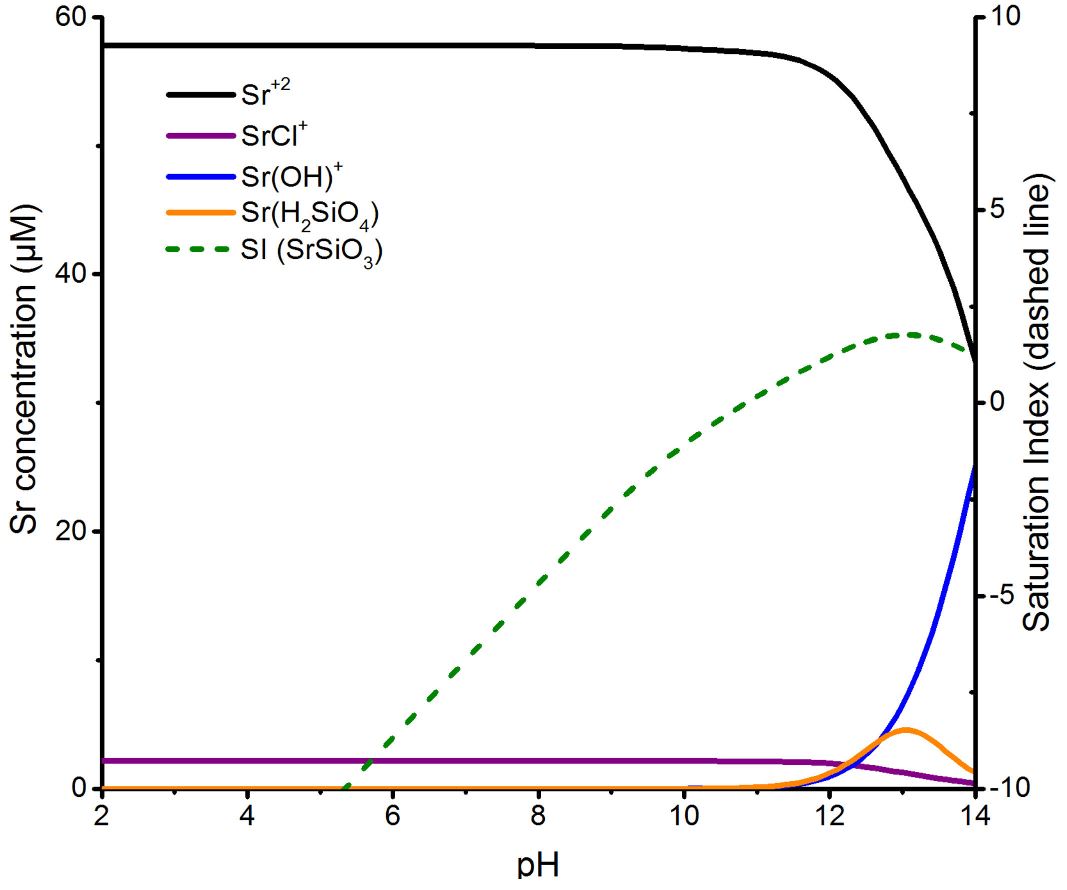

Figure S7: Thermodynamic modelling of Sr speciation and saturation index of strontium metasilicate, $SrSiO_3$, indicating that Sr is oversaturated at pH 12 and 14 with respect to $SrSiO_3$.

EXAFS fitting

Table S1: Full list of first shell Sr-O, Sr-Si and Sr-Sr interatomic distances for $SrSiO_3$ (Nishi, 1997)

Path	N	R(Å)
Sr-O.1	2	2.46
Sr-O.2	4	2.69
Sr-O.3	2	2.74
Sr-Si.1	4	3.34
Sr-Si.2	2	3.87
Sr-Sr.1	2	4.02
Sr-Sr.2	1	4.05
Sr-Sr.3	2	4.16
Sr-Sr.4	1	4.30

Table S2: EXAFS fits for Sr/U(IV)-silicate system at pH 12 and pH 14 showing fits to Sr-U-Si coordination environment. In both cases, R value (goodness of fit) is significantly higher than for Sr-silicate fitting (Table 2), indicating lower quality of fit.

Sample	Path	N	R (Å)	σ^2	ΔE_0	R
**pH 12**	**Sr-O**	9	2.61(1)	0.009(5)	0.5 (7)	0.0088
	**Sr-Si**	1.8	3.59(5)	0.013(7)		
	**Sr-U**	1.8	3.75(4)	0.012(5)		
**pH 14**	**Sr-O**	9	2.60(1)	0.010(1)	0.4(10)	0.027
	**Sr-Si**	1.8	3.46(3)	0.012(5)		
	**Sr-U**	1.7	3.64(4)	0.015(5)		

Coordination numbers (N), U bond distances (R (Å)), Debye-Waller factors (σ^2), shift in energy from calculated Fermi level (ΔE_0) and 'goodness of fit' factor (R). Coordination numbers were fixed, amplitude factors were fixed as 1. Numbers in parentheses are the standard deviation on the last decimal place.

References

Fuller, A. J.; Shaw, S.; Peacock, C. L.; Trivedi, D.; Burke, I. T., EXAFS Study of Sr sorption to Illite, Goethite, Chlorite, and Mixed Sediment under Hyperalkaline Conditions. Langmuir **2016,** 32 (12), 2937-2946.

Sureda, R.; Martínez-Lladó, X.; Rovira, M.; de Pablo, J.; Casas, I.; Giménez, J., Sorption of strontium on uranyl peroxide: Implications for a high-level nuclear waste repository. Journal of Hazardous Materials **2010,** 181 (1-3), 881-885.

Wieland, E.; Tits, J.; Kunz, D.; Dähn, R., Strontium uptake by cementitious materials. Environmental Science and Technology **2008,** 42 (2), 403-409.
